# Preclinical Evaluation of a B7-H3 Targeting Antibody Enhancing NK Cell-Mediated Cytotoxicity for Ovarian Cancer Treatment

**DOI:** 10.2147/ITT.S521008

**Published:** 2025-07-11

**Authors:** Ilona Hagelstein, Sven Mattern, Kevin Wang, Yannick E Haueisen, Sarah M Greiner, Alexander Englisch, Annette Staebler, Stephan Singer, Martina S Lutz

**Affiliations:** 1 German Cancer Consortium (DKTK), Partner Site Tuebingen, a Partnership Between DKFZ and University Hospital Tuebingen, Tuebingen, Germany; 2 Clinical Collaboration Unit Translational Immunology, Department of Internal Medicine, University Hospital Tübingen, Tuebingen, Germany; 3 Cluster of Excellence iFIT (EXC 2180) Image-Guided and Functionally Instructed Tumor Therapies, University of Tuebingen, Tuebingen, Germany; 4 Department of Pathology and Neuropathology, University Hospital Tuebingen, Tuebingen, Germany; 5 Department of Obstetrics and Gynecology, University Hospital Tuebingen, Tuebingen, Germany; 6 Department of Peptide-Based Immunotherapy, Institute of Immunology, University and University Hospital Tuebingen, Tuebingen, Germany

**Keywords:** B7-H3, monoclonal antibody, ovarian cancer, NK cell, immunotherapy

## Abstract

**Background:**

Despite the advancements in treatment, ovarian cancer remains the deadliest gynecological malignancy. The dismal prognosis of the disease necessitates the urgent development of novel therapies. Monoclonal antibodies (mAbs) have transformed cancer treatment, yet their effectiveness in ovarian cancer remains limited. A key mechanism in mAb therapy is antibody-dependent cellular cytotoxicity (ADCC), driven by natural killer (NK) cells targeting tumor cells. Optimization of the Fc domain of mAbs to enhance efficacy has therefore become a subject of extensive research. The costimulatory molecule B7-H3 is overexpressed in various cancers, including ovarian cancer, making it a promising target for anti-tumor mAb immunotherapy. This study evaluates the preclinical potential of an Fc-optimized B7-H3-targeting antibody for ovarian cancer treatment.

**Methods:**

The expression of B7-H3 was evaluated in tumor samples from 43 ovarian cancer patients using immunohistochemistry. A chimeric B7-H3 mAb was developed with a wildtype Fc (8H8-WT) and an Fc-optimized variant (8H8-SDIE) containing S239D/I332E substitutions to enhance CD16 binding and subsequent activation of NK cells. The therapeutic effects of 8H8-SDIE were evaluated through NK cell activation, cytokine release, and cytotoxicity assays.

**Results:**

A total of 43 ovarian cancer samples were analyzed, and it was found that all of them expressed B7-H3. In addition, 8H8-SDIE was found to demonstrate significantly higher affinity for CD16 than 8H8-WT, with minimal effects on other Fc receptors. Functional assays confirmed that 8H8-SDIE enhanced NK cell activation and promoted IFN-γ and TNF release. Furthermore, 8H8-SDIE induced robust cytotoxicity against B7-H3-expressing ovarian cancer cells in both short-term and long-term assays.

**Conclusion:**

8H8-SDIE has been shown to induce potent NK cell activity, resulting in tumor cell lysis. This finding underscores its promise as an innovative immunotherapeutic approach for the treatment of ovarian cancer.

## Graphical Abstract

**Figure:**
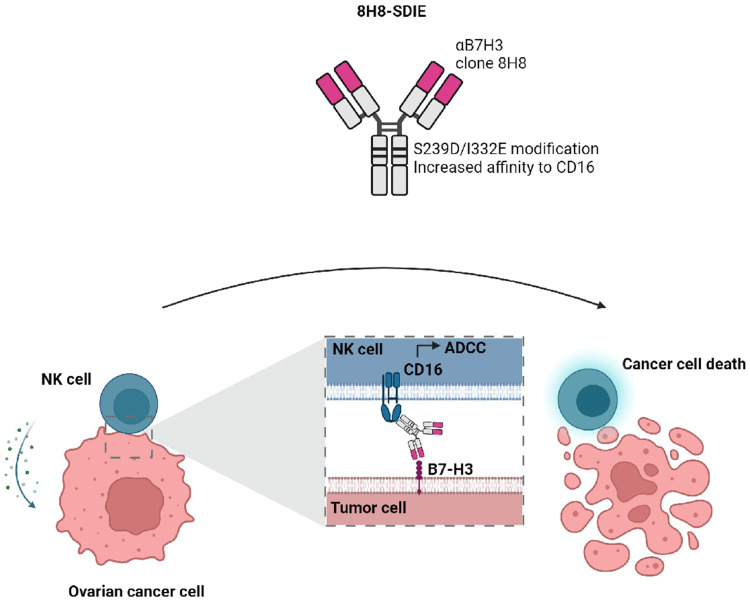

## Introduction

Ovarian cancer constituted the eighth most frequently diagnosed neoplasm in females and the third most commonly occurring gynecological malignancy globally in 2020.^1^,^2^ It is classified into five major histologic subtypes, with high-grade serous carcinoma (HGSC) being the most prevalent.^3^ Standard of care primary treatment involves surgery and platinum-based chemotherapy.^3^,^4^ Advanced-stage patients also receive bevacizumab, a monoclonal antibody (mAb) targeting vascular endothelial growth factor (VEGF). Patients with BRCA1/2 mutations or other homologous recombination deficiencies (HRD) benefit from maintenance therapy with PARP inhibitors. Despite these advancements, the prognosis for ovarian cancer remains poor, with a 17% 5-year survival rate for advanced-stage patients. This phenomenon can be attributed to the following factors: firstly, the diagnosis is often delayed, and secondly, the disease recurs frequently after primary treatment.^5^ Due to a lack of specific early symptoms, ovarian cancer is often diagnosed at an advanced stage, making long-term disease control challenging.^6^ While the majority of patients initially respond well to platinum-based chemotherapy, recurrence is common, and prognosis significantly worsens once platinum resistance develops.^7^ Patients with platinum-sensitive relapse may benefit from platinum rechallenge; however, therapeutic options become more limited with subsequent relapses. Poly-ADP-ribose polymerase (PARP) inhibitors have shown substantial benefit, particularly in patients with HRD and BRCA mutations.^8^,^9^ Although resistance to PARP inhibitors can develop over time, data from trials such as SOLO1 suggest that PARP inhibitor maintenance therapy has the potential to improve long-term outcomes and may even lead to cure in a subset of patients.^10^ Despite these advances, the high relapse rate and eventual resistance to current therapies highlight the ongoing need for novel treatment strategies.

In recent years, regulatory approval has been granted for several immunotherapeutic modalities for both hematologic malignancies and solid tumors. These therapeutic options include anti-tumor antibodies, antibody-drug-conjugates (ADCs), immune checkpoint blockade, adoptive cell therapy, and cancer vaccines.^11^ Inspired by progress in other gynecologic cancers like endometrial cancer or cervical cancer, immunotherapeutic strategies have gained interest also for ovarian cancer.^12^ However, aside from the angiogenesis inhibitor bevacizumab,^13–15^ few immunotherapeutic antibodies have obtained approval for use in the treatment of ovarian cancer, primarily in the context of recurrent disease. Mirvetuximab soravtansine, an ADC, is approved for patients with folate receptor alpha positive ovarian cancer.^16^,^17^ Trastuzumab deruxtecan, the first tumor-agnostic ADC, is approved for use in tumors with high HER2 expression.^18^ The KEYNOTE-158 trial led to the approval of the checkpoint inhibitor pembrolizumab in deficient mismatch repair (dMMR), microsatellite instability-high (MSI-H), or high tumor mutational burden (TMB-H) solid malignancies and, therefore, is approved in patients with recurrent ovarian cancer with these characteristics, which however are uncommon in HGSC.^19^ Furthermore, the efficacy of checkpoint inhibitors in the treatment of ovarian cancer remains to be demonstrated.^20^,^21^

Antibody-based immunotherapies have become a powerful treatment option in cancer therapy and have markedly improved the outcomes in many malignancies. Anti-tumor antibodies include mAbs like rituximab or tafasitamab for the treatment of hematologic malignancies,^22^,^23^ and trastuzumab for the treatment of HER2-positive breast cancer.^24^ Bispecific antibodies (bsAbs) like blinatumomab or mosunetuzumab are also used in B cell malignancies.^25^,^26^

The primary effector cells activated by mAbs are natural killer (NK) cells. NK cell functions are controlled by a variety of activating and inhibitory receptors.^27^ In the context of mAbs, the activating Fc gamma receptor IIIa (FcγRIIIa), also known as CD16, has a key function in initiating activation signals in NK cells upon binding to mAb Fc domains. This results in antibody-dependent cellular cytotoxicity (ADCC) which induces NK effector functions and subsequently results in tumor cell lysis.^28^ To improve the capability of mAbs to recruit NK cells and thus target and eradicate tumor cells, there are several strategies aimed at boosting the Fc-mediated functions.^29^ One promising approach is the introduction of amino acid mutations into the Fc part such as the S293D/I332E (SDIE) modification to selectively increase the affinity for FcγRIIIa.^30^

These mAbs are supposed to target so called tumor-associated antigens, which are almost exclusively overexpressed on tumor cells, allowing for specific targeting of these cells. One of the target antigens currently under extensive investigation is B7-H3 (CD276).^31^ The role of the B7-family members of costimulatory and coinhibitory ligands in tumor evolution is not yet precisely understood.^32^ However, recent reports have linked its expression in various cancers,^33^,^34^ including ovarian cancer, with poor outcomes and a higher risk of relapse for patients.^35^,^36^ Mechanistically, B7-H3 has been shown to suppress T cell proliferation and cytokine production,^37^,^38^ as well as to inhibit NK cell-mediated cytotoxicity.^39^ In head and neck squamous cell carcinoma, cancer stem cells have been found to exploit B7-H3 to escape immune surveillance.^40^ Moreover, B7-H3 is expressed in most solid tumors with consistent and elevated cell surface levels. It has been demonstrated that B7-H3 expression in ovarian cancer occurs independently of the status of the BRCA mutation,^41^ suggesting its potential as a broadly applicable therapeutic target for both HRD-positive and HRD-negative ovarian cancer patients. Additionally, the expression of B7-H3 in ovarian cancer includes the tumor surrounding structures such as the tumor vasculature and the tumor stroma.^35^,^41^,^42^

We have recently developed and introduced an Fc-optimized mAb (B7-H3-SDIE) that demonstrated improved NK cell reactivity against sarcomas, pancreatic cancer and acute myeloid leukemia compared to the Fc wildtype version bearing the same B7-H3 binder.^43–45^ Despite the mentioned recent advances in the application of anti-tumor mAbs, a major gap in ovarian cancer treatment is the lack of antibodies that redirect immune cells against tumor cells. Bevacizumab inhibits angiogenesis, and pembrolizumab acts as an immune checkpoint inhibitor, but neither directly targets tumor cells for immune recognition. ADCs like mirvetuximab soravtansine and trastuzumab deruxtecan rely on their cytotoxic payloads, causing toxicity. These agents, while progress, do not enhance the immune system’s ability to recognize and clear tumor cells.

With the final aim to provide an immune system engaging, highly tumor-specific therapeutic treatment options for affected women, we report on the preclinical study of our B7-H3-targeted, Fc -optimized mAb for ovarian cancer patients.

## Materials and Methods

### B7-H3 Expression in Ovarian Cancer Tissues

Freshly sectioned patient derived ovarian cancer tissues (n = 43 patient cases) were fixed in formalin and embedded in paraffin (FFPE). For each patient, two distinct tissue specimens with varying intraabdominal localizations were included. Tumor tissues were obtained from the biobank of the Institute of General and Molecular Pathology and Pathological Anatomy Tuebingen. For tissue microarray (TMA) preparation, three pieces were cored from each FFPE block (diameter 0.6 mm) and assembled into tissue microarrays, leading to six samples per patient from two distinct tissues. Due to technical issues affecting a subset of the cores, these samples were excluded from the analysis, resulting in a total of five to six samples per patient, with two to three samples per tissue origin from each patient. Immunohistochemical staining was performed on 2.5 micron sections of the TMA using the B7-H3 antibody RBT-B7H3 (Medac/Bio SB) on an automated VENTANA BenchMark ULTRA (Roche, Basel, Switzerland) according to the manufacturer’s protocol. To confirm the ovarian cancer diagnosis, the sections were stained with hematoxylin and eosin (H&E). The staining intensity of B7-H3 was evaluated under the supervision of a board-certified pathologist (S.M.) using a standard scoring system, where 0 indicates no staining, 1 indicates weak but detectable staining, 2 indicates moderate but clearly positive staining, and 3 indicates strong staining.^33^ Additionally, two independent evaluations were conducted to assess the expression of B7-H3. The staining of the tumor stroma and tumor vessels was considered to be positive when the staining score was 1 or above. To quantify the degree of tumor cell staining, the H-scores were calculated as follows:^46^

$${\mathrm{H}} - {\mathrm{Score}} = \mathop \sum \limits_{{\mathrm{n}} = 0}^3 \left({{\mathrm{n*percentage\,of\,cells\,with\,staining\,intensity\,n}}} \right)$$

### Cells

The cell lines OVCAR-3, OVCAR-4, OVCAR-5, OVCAR-8 and NCI/ADR-RES were obtained from the American Type Culture Collection (ATCC) and maintained in Gibco DMEM (Thermo Fisher Scientific, Waltham, MA) supplemented with 10% FBS (Capricorn, Ebsdorfergrund, Germany) and 1% Penicillin-Streptomycin (PAN-Biotech, Aidenbach, Germany). A working cell bank was established for all cell lines used in this study, with the number of passages defined. The cells were maintained in culture in for a maximum period of eight weeks. Mycoplasma contamination was checked every month. Peripheral blood mononuclear cells (PBMC) were isolated from healthy donors of various ages and genders using density gradient centrifugation (Biochrom, Berlin, Germany). PBMC samples were randomly selected for each experiment after overnight culturing at 37°C. Prior to functional experiments, cryopreserved cells were thawed and cultured in media. Where indicated, NK cells within PBMC were isolated using a NK cell isolation kit (Miltenyi Biotec, Bergisch Gladbach, Germany) for isolating untouched NK cells. In brief, PBMC were labelled with the NK Cell Biotin-Antibody Cocktail, followed by a NK Cell MicroBead Cocktail. Subsequently, NK cells were isolated by Magnetic-activated cell sorting. Purity of the isolated NK cells was analyzed by flow cytometric staining for CD3 and CD56 and was at least 95%.

### Polymerase Chain Reaction (PCR)

To determine B7-H3 mRNA levels, B7-H3 primers from the QuantiTect Primer Assay Hs_CD276_1_SG (Qiagen) were used, while RPL13 (Hs_RPL13_1_SG, Qiagen) was used as the housekeeping gene. Total RNA was isolated from 1–2 x 10^6^ cells using the High Pure RNA Isolation Kit (Roche, Basel, Switzerland), followed by cDNA synthesis with FastGeneScriptase II (NIPPON Genetics Europe, Dueren, Germany) according to the manufacturer’s instructions. The previously described methods were used to conduct reverse transcription-polymerase chain reaction (RT-PCR).^47^,^48^ For quantitative PCR (qPCR), the PerfeCTa SYBR Green FastMix (Quanta Biosciences, Beverly, MA) was used with a LightCycler 480 instrument from Roche. Relative B7-H3 mRNA expression—normalized to RPL13 RNA—was calculated by the ΔΔ cycle-threshold (Ct) method.

### Antibody Production and Purification

The B7-H3 targeting mAb with a wildtype Fc part (8H8-WT), with an optimized Fc part (8H8-SDIE) and the corresponding isotype control (MOPC-SDIE) were chimerized by fusing the B7-H3 mAb (clone 8H8) and a control mAb (clone MOPC21) with the human immunoglobulin G1/κ constant region.^43^ The mAbs were Fc-optimized using the S239D/I332E modification as described previously.^49^ The plasmids encoding the light and heavy chains of the mAbs were obtained using the EndoFree Plasmid Maxi kit from Qiagen. The antibodies were produced using the ExpiCHO cell system from Gibco. Cells were cultured in the HyClone Trans FX-C medium (Cytiva, Marlborough, MA) supplemented with 40 mM L-glutamine (Thermo Fisher scientific) and 3 mM Pluronic (Thermo Fisher scientific), maintained at 37 °C and 8% CO₂. Transfection was carried out after six days of cell expansion, at a density of 1 × 10⁶ cells/mL, using 0.5 µg of DNA per mL of transfection volume in a 3:2 ratio of heavy chain to light chain plasmid. Polyethylenimine (PEI; Polysciences, Warrington, PA) was used as the transfection reagent (PEI:DNA ratio of 6:1). One day post-transfection, culture conditions were adjusted to 32 °C and 5% CO₂, and feeding was performed on days 1, 4, and 6 using Feed A and Feed B (Cytiva). Supernatants were harvested seven days after transfection. Subsequent purification of the antibodies from the media was conducted through protein A affinity chromatography (GE Healthcare, Chicago, IL), followed by preparative size exclusion chromatography using a HiLoad 16/60 Superdex 200 column (GE Healthcare). To guarantee the quality and purity of the antibodies produced, we conducted analytical size exclusion chromatography using Superdex 200 Increase 10/300 GL columns from GE Healthcare, along with 4–12% gradient SDS-PAGE gels (Invitrogen, Carlsbad, CA) with the Precision Plus standard (Bio-Rad, Hercules, CA). To ensure endotoxin levels below 0.5 EU/mL, mAb solutions were tested with an EndoZyme assay (Biomerieux, Nuertingen, Germany).

### Fc Gamma Receptor (FcγR) ELISA

An ELISA-based FcγR binding analysis was conducted using immobilized FcγRI, FcγRIIa, FcγRIIb, FcγRIIIa (158V variant) and FcγRIIIb (all from BioLegend, San Diego, CA). After blocking, mAbs were added subsequently to the plate at the indicated concentrations, and binding was visualized using an HRP-conjugated goat anti-human-Fc antibody (Jackson ImmunoResearch, West Grove, PA).

### Flow Cytometry

The surface expression of B7-H3 was assessed using a fluorescence-conjugated (PE-Cy7) mAb for B7-H3 or an isotype control (BioLegend). For dose titration and binding experiments, cells were first stained with 8H8-SDIE or MOPC-SDIE, followed by an anti-human phycoerythrin (PE) conjugate (Jackson ImmunoResearch). A quantitative analysis of immunofluorescence was conducted to determine the number of B7-H3 molecules present on the cell surface using a murine B7-H3 hybridoma-derived antibody and the QIFIKIT from Dako (Hamburg, Germany), following established procedures.^47^,^48^

To evaluate NK cell activation and degranulation, cells were stained with fluorescence-labelled antibodies. These included CD3-APC/Fire, CD56-PECy, CD16-APC, CD25-BV711, and CD69-PE (all from BioLegend), as well as CD107a-PE (BD Biosciences, Franklin Lakes, NJ).

To exclude dead cells from flow cytometric analysis, either 7-AAD (BioLegend) staining (1:200) or LIVE/DEAD™ Fixable Aqua (Thermo Fisher Scientific) was used. Sample analysis was performed using either the BD FACS Canto II or BD Fortessa (BD Biosciences). Data analysis was carried out with FlowJo software (FlowJo LCC, Ashland, OR).

### NK Cell Activation and Degranulation Assays

To determine NK cell activation in the absence of tumor cells, mAbs (1 µg/mL) were immobilized on plastic, followed by a blocking step (medium + 10% FBS). Then, PBMC (200,000 cells/well) were added and analyzed after 24 and 72 hours for expression of the activation markers CD69 and CD25 by flow cytometry.

To determine NK cell activation and degranulation in the presence of tumor cells, the latter were cultured with PBMC at an effector-to-target (E:T) ratio of 2.5:1 with or without the indicated treatment (1 µg/mL). Brefeldin A (GolgiPlug, BD Biosciences) was added for analysis of degranulation. After 4 hours, cells were stained for CD107a expression and analyzed by flow cytometry. To determine NK cell activation, the coculture was stained for CD69 and CD25 expression after 24 and 72 hours.

### Image Stream

Tumor cells were incubated with isolated NK cells (E:T 1:1) for 45 minutes, with or without 8H8-SDIE or MOPC-SDIE. After incubation, cells were stained with a FITC-labelled anti-human Fc-specific antibody (Jackson ImmunoResearch) to detect cell-bound antibodies. The cells were then surface-stained with CD56-PECy7, CD16-BV605, and CD326-PE (all from BioLegend) and subsequently fixed in 1.5% paraformaldehyde (PFA) in PBS for 15 minutes at room temperature (RT). The cells were washed with PBS and then incubated with PBS-T (PBS + 0.1% Tween20) for 20 minutes at RT. Next, DAPI (0.1 µg/mL, Invitrogen) and Phalloidin-AF647 (1:500, Thermo Fisher Scientific) were added to stain the nuclei and actin, respectively. The amnis ImageStream mkII Imaging Flow cytometer (Cytek Biosciences, San Diego, CA) was used to collect at least 100,000 cells at 40x magnification. The data were analyzed using IDEAS^®^ Image analysis software. The samples were gated on binuclear aggregates. The identification of organized immunological synapses was based on the measurement of colocalization between two images, one of single tumor cells and the other of single NK cells, in the overlapping region of polarized Phalloidin.

### Analysis of Cytokine Secretion

PBMC were cultured with or without tumor cells (E:T 2.5:1) in the presence or absence of the indicated mAbs (1µg/mL) for 4 hours. Subsequently, supernatants were harvested and analyzed for TNF and IFN-γ using Legendplex assays (BioLegend).

### Cytotoxicity Assays

The cytotoxic activity of PBMC against tumor cells was assessed through BATDA Europium assays (PerkinElmer, Waltham, MA) conducted over a 2-hour period, following established protocols. The percentage of specific lysis was determined using the following formula:

$${\mathrm{specific\,lysis}} = {\mathrm{}}100{\mathrm{}} \times {\mathrm{}}{{\left[{\left({{\mathrm{experimental\,release}}} \right){\mathrm{}} - {\mathrm{}}\left({{\mathrm{spontanous\,release}}} \right)} \right]} \over {\left[{\left({{\mathrm{maximum\,release}}} \right){\mathrm{}} - {\mathrm{}}\left({{\mathrm{spontanous\,release}}} \right)} \right]}}$$

To evaluate target cell lysis using flow cytometry-based assays, tumor cells were loaded with 2.5 µM CellTrace^TM^ Violet (Thermo Fisher Scientific) and cultured with PBMC at an E:T of 10:1 in the presence or absence of 8H8-SDIE or MOPC-SDIE (1 µg/mL). To ensure consistency in test volumes and to standardize the assay volume and the count of viable target cells, standard calibration beads (Sigma-Aldrich, St. Louis, MO) were employed.

To conduct long-term cytotoxicity analyses, we used the xCELLigence RTCA system (Roche Applied Science, Penzberg, Germany). Tumor cells were cultured with PBMC at an E:T ratio of 10:1, both with and without the specified mAbs (1 µg/mL each). Cell impedance was monitored in real-time at 30-minute intervals over 120 hours and expressed as cell Index. Data analysis was performed using the RTCA Software, and area under the curve (AUC) values were calculated to quantify the overall cytotoxic response over the full duration of the experiment. Cell index values were normalized to the time point at which PBMCs and monoclonal antibodies were added.

### Statistics

Unless otherwise specified, values are presented as means ± standard deviation (SD). Statistical analysis was performed using GraphPadPrism (v.8.1.0) and included one-way ANOVA, and Kruskal–Wallis test for continuous variables. If significant differences were found by ANOVA, group-wise comparison was performed using Tukey’s multiple comparison test. If significant differences were found by Kruskal–Wallis test, Dunn’s multiple comparisons test was used. All statistical tests were considered statistically significant when *p* was below 0.05.

## Results

### B7-H3 Expression in Ovarian Cancer

**Figure 1:**
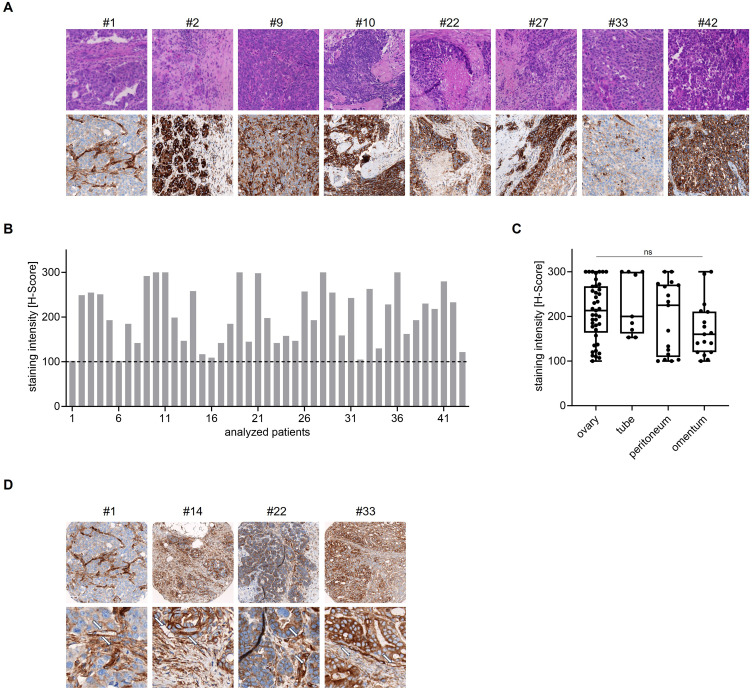
B7-H3 expression in tumor tissues from ovarian cancer patients. Immunohistochemical analysis of B7-H3 surface expression on formalin fixed and paraffin-embedded (FFPE) patient derived ovarian cancer tissues. Staining intensity scores were assessed under the supervision of a board-certified pathologist (0 = no staining, 1 = weak but detectable staining, 2 = moderate but clearly positive staining, and 3 = strong staining). (**A**) Representative images of tumor tissue sections after hematoxylin-eosin (H&E) and B7-H3 staining taken from tissue microarray (20x magnification). (**B** and **C**) Quantification of B7-H3 expression intensities by H-scores in n = 43 patient cases. The H-Score of a given sample represents the summed multiplicates for each staining intensity score with their respective proportionate prevalence of all tumor cells in percent. Dotted line represents threshold value for positivity. (**B**) Mean H-Score of all combined samples from each patient pooled from two distinct tissue origins with varying intraabdominal localizations. Depending on sample quality 5–6 samples were included per patient. (**C**) Mean H-Score determined individually for indicated origin of each tissue with 2–3 samples each per patient. (**D**) Exemplary images of tumor tissue sections after B7-H3 staining. The upper panel displays images with 10x magnification, while the lower panel presents images with 40x magnification of the same patient sample. The white arrows depict tumor surrounding structures including stroma, vessels and capillaries. ns = not significant (p > 0.2).

HGSC represents the most prevalent form of ovarian cancer, accounting for approximately 70–80% of epithelial ovarian cancers.^50^ Consequently, patient derived tissue sections from 43 HGSC patients were immunohistochemically analyzed for B7-H3 expression. The clinical characteristics of the 43 ovarian cancer patients included in the study are summarized as follows: the mean age was 63.7 years (range: 34–90). Most tumors were classified as T3 (84%) and N1 (56%), with 14% of patients presenting with distant metastases (M1). The majority were diagnosed at FIGO stage III (74%) or IV (14%). Primary debulking surgery was performed in 86% of cases, and 63% of patients received adjuvant chemotherapy with a combination of carboplatin and paclitaxel. A smaller subset received neoadjuvant chemotherapy or alternative treatment regimens (Supplemental Table 1, Table 1). H&E staining was used to confirm histopathological diagnosis and tumor localization within the TMA samples. Two distinct intraabdominal tumor localizations per patient are illustrated, providing a visual overview of the staining patterns observed across the cohort (Supplemental Figure S1). Immunohistochemical analysis revealed a consistent membranous expression of B7-H3 on ovarian cancer tumor cells (Figure 1A). All analyzed tumor samples were positive for B7-H3 expression with H-scores above the threshold value for positivity (H-score = 100) (Figure 1B and Supplemental Table 2). Consistent B7-H3 expression was observed in our analyzed patient cohort irrespective of the intraabdominal tumor localization sites including the tube, ovary, omentum, or peritoneum (Figure 1C, Supplemental Tables 2 and 3). In addition, the expression of B7-H3 was observed to encompass the tumor surrounding structures including stroma, vessels and capillaries (Figure 1D).

**Table 1:** Patient characteristics

Clinical Characteristics	Total (n = 43)
**Age**	
Age in years, mean-yr, ± SD (range)	63.7 ± 14.2 (34–90)
**TNM classification, n (%)**	
**Stage**	
T0	0 (0)
T1	3 (7)
T2	3 (7)
T3	36 (84)
T4	0 (0)
Unknown	1 (2)
**Node**	
NX	4 (9)
N0	15 (35)
N1	24 (56)
Unknown	0 (0)
**Metastasis**	
M0	30 (70)
M1	6 (14)
Unknown	7 (16)
**FIGO classification, n(%)**	
1	2 (5)
2	2 (5)
3	32 (74)
4	6 (14)
Unknown	1 (2)
**Treatment, n (%)**	
**Neoadjuvant chemotherapy**	
Carboplatin	1 (2)
Unknown	5 (12)
**Surgery**	
Primary debulking surgery	37 (86)
Unknown	5 (12)
**Adjuvant chemotherapy**	
Carboplatin	5 (12)
Carboplatin + Paclitaxel	27 (63)
Carboplatin + other	1 (2)
Other	1 (2)
Unknown	5 (12)

**Figure 2:**
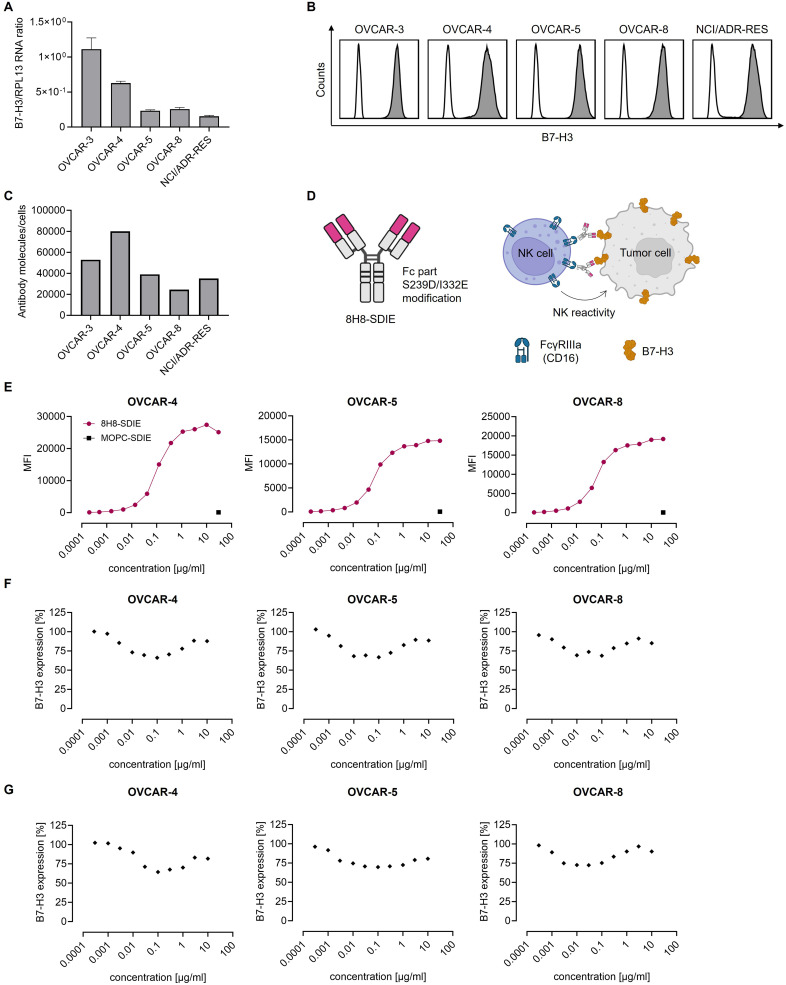
Expression of B7-H3 and binding profile of 8H8-SDIE in ovarian cancer cells. (**A**) Relative RNA levels of B7-H3 for ovarian cancer cell lines (OVCAR-3, OVCAR-4, OVCAR-5, OVCAR-8 and NCI/ADR-RES) for a total of n = 3 experiments are shown. (**B**) Expression of B7-H3 on the indicated ovarian cancer cells was analyzed by flow cytometry. Indicated cells were stained with a monoclonal B7-H3 antibody (clone 7C4) (shaded peaks) or isotype control (open peaks) followed by an anti-mouse phycoerythrin (PE) conjugate. (**C**) B7-H3 molecules per cell were determined by QIFIKIT on ovarian cancer cell lines by flow cytometry. (**D**) The B7-H3-targeting antibody 8H8-SDIE contains a human IgG1 Fc with amino acid substitutions (SDIE modification) enhancing its affinity to FcγRIIIa (CD16) (left panel). Binding of 8H8-SDIE to B7-H3 on ovarian cancer cells induces NK cell reactivity. Created with BioRender.com. (**E**) The indicated ovarian cancer cells were stained with depicted concentrations of 8H8-SDIE or MOPC-SDIE followed by an anti-human PE conjugate and analyzed by flow cytometry. (**F** and **G**) Ovarian cancer cells were incubated with the indicated concentration of 8H8-SIDE for (**F**) 24 hours or (**G**) 72 hours. Cells were then washed and directly reincubated with 8H8-SDIE followed by an anti-human PE antibody and measured by flow cytometry. Relative surface expression of B7-H3 was calculated by defining the mean fluorescence intensity (MFI) of cells preincubated without 8H8-SDIE as 100%. Shown are representative results out of three experiments.

Following confirmation of B7-H3 expression in patients, expression was also analyzed in the ovarian cancer cell lines OVCAR-3, OVCAR-4, OVCAR-5, OVCAR-8, and NCI/ADR-RES. Analyses of mRNA levels revealed differing B7-H3 expression levels across the examined cell lines (Figure 2A). Given the pivotal role of target antigen expression on the cell surface for antibody-based immunotherapy, a flow cytometric analysis was conducted, which demonstrated B7-H3 cell surface expression for all cell lines tested (Figure 2B). The range of B7-H3 surface molecules exhibited considerable variation, with values spanning from 24,543 per cell (OVCAR-8) to 80,050 per cell (OVCAR-4) (Figure 2C). OVCAR-5 cells (39,103 molecules per cell) were selected for further analysis as a representative of moderate B7-H3 expression, slightly lower than OVCAR-3 (52,993 molecules/cell). NCI/ADR/RES (35,059 molecules/cell) were not chosen to avoid redundancy, because the cell lines OVCAR-8 and NCI/ADR-RES are derived from the same individual.^51^ Based on these findings, OVCAR-4, OVCAR-5, and OVCAR-8 were selected as high-, middle-, and low-expressing cell lines, respectively, for subsequent functional analyses. From a panel of mAbs directed to B7-H3 (described in patent application EP3822288A1), a high-affinity, chimeric mAb (clone 8H8) was selected for generation of our B7-H3-targeting mAbs. The mAbs were produced in two forms: a human IgG1 wild-type (8H8-WT) and a human IgG1 variant in which amino acid substitutions S239D/I332E (8H8-SDIE) were introduced. These substitutions are known to enhance the affinity of the mAb to the FcγRIIIa, which is a key mediator of ADCC (Figure 2D). Dose titration experiments, conducted at concentrations ranging from 0.0002 to 30 µg/mL, demonstrated that 8H8-SDIE binding reached saturation at a concentration of 1 µg/mL (Figure 2E). This concentration was subsequently employed in functional analyses. The binding of an antibody to its target molecule frequently results in a dose-dependent modulation of target antigen expression. This, in turn, impairs the efficacy of the therapeutic intervention.^52^ To assess the induction of this phenomenon known as antigen shift, we examined B7-H3 expression after incubating ovarian cancer cells with 8H8-SDIE (range 0.0003–10 µg/mL) for 24 and 72 hours. We observed only a slight decrease (maximum of 30% reduction) in B7-H3 surface expression (Figure 2F and G).

### SDIE Modification Increases CD16 Binding and NK Cell Activation

**Figure 3:**
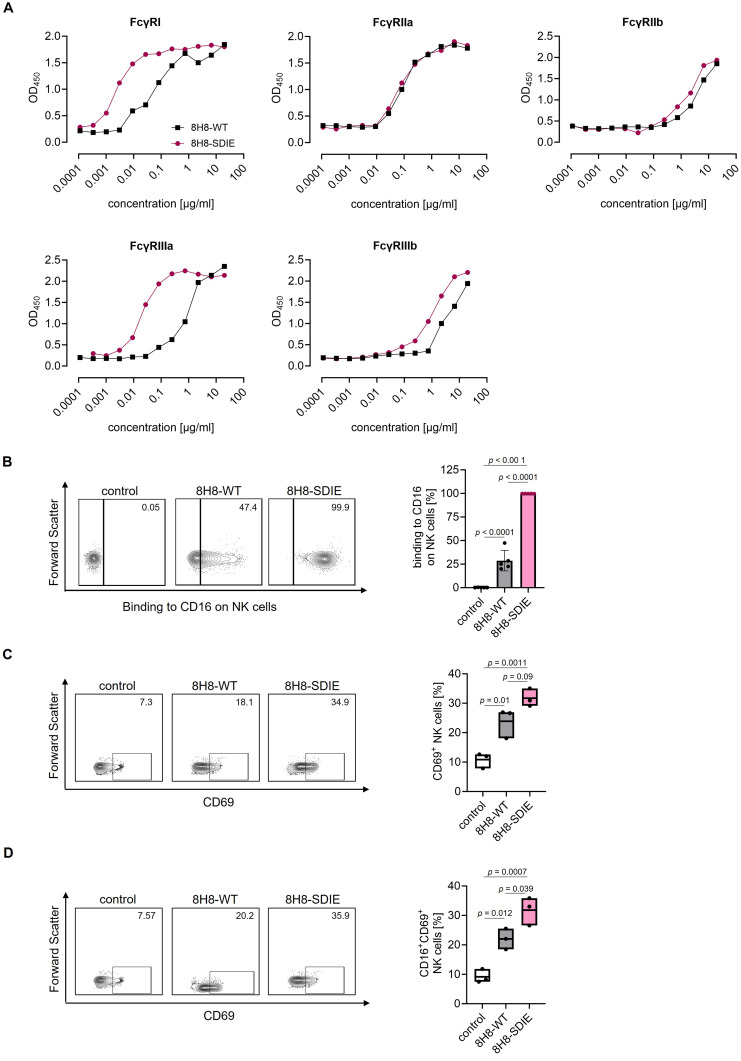
Enhanced Fc-receptor binding and effector functions of SDIE modified mAb 8H8-SDIE vs 8H8-WT. (**A**) Binding of 8H8-SDIE (Fc-optimized) and 8H8-WT (wild type Fc part) to the indicated FcγR proteins was determined by ELISA. Exemplary results out of three experiments are shown. (**B**) Binding of 8H8-SDIE and 8H8-WT to CD16 (FcγRIIIa) on NK cells (CD56^+^/CD3^−^) within resting PBMC was analyzed by flow cytometry. PBMC were incubated with or without (control) the indicated mAbs followed by anti-human-PE. Left panels depict exemplary results for one PBMC donor, right panel shows combined results for a total of five donors. Statistical significance was calculated by one-way ANOVA and Tukey’s multiple comparisons test. (**C** and **D**) PBMC were incubated for 72 hours on the indicated mAbs immobilized to plastic (1 µg/mL) or without mAbs (control), followed by determination of the activation marker CD69 on (**C**) NK cells (CD56^+^/CD3^−^) or on (**D**) CD16^+^ NK cells (CD16^+^/CD56^+^/CD3^−^) by FACS. Left panels show representative results of single experiments, right panels show results obtained with PBMC of three independent donors. Box plots with mean ± SD. Statistical significance was calculated by one-way ANOVA and Tukey’s multiple comparisons test.

To ascertain the extent to which the SDIE modification affects the affinity of our mAbs for different Fc receptor subtypes, FcγR ELISAs were conducted. The binding analysis of our 8H8-SDIE revealed a substantial increase in affinity to FcγRIIIa in comparative analysis with its Fc wild-type counterpart. Additionally, an increased affinity to FcγRI was observed, while minimal effects on other Fc receptor subtype binding affinities were noted (Figure 3A). Subsequently, we analyzed the binding of both mAbs to CD16 (FcγRIIIa) on NK cells within resting PBMC. The 8H8-SDIE exhibited markedly enhanced binding to NK cells in comparison to the 8H8-WT (Figure 3B). As a next step, we sought to determine whether the SDIE modification enhances NK cell activation. To this end, PBMC were incubated on coated mAbs and subsequently analyzed for CD69 expression. Treatment with 8H8-SDIE induced a more robust NK cell activation than 8H8-WT (Figure 3C). This finding was also observed when analyzing activation of CD16⁺ NK cells within PBMC (Figure 3D). We concluded that the 8H8-SDIE exhibits markedly enhanced binding affinity for FcγR CD16, thereby demonstrating an amplified capacity to activate NK cells.

### 8H8-SDIE Modulates NK Cell Activation and Synapse Formation Against Ovarian Cancer Cells

**Figure 4:**
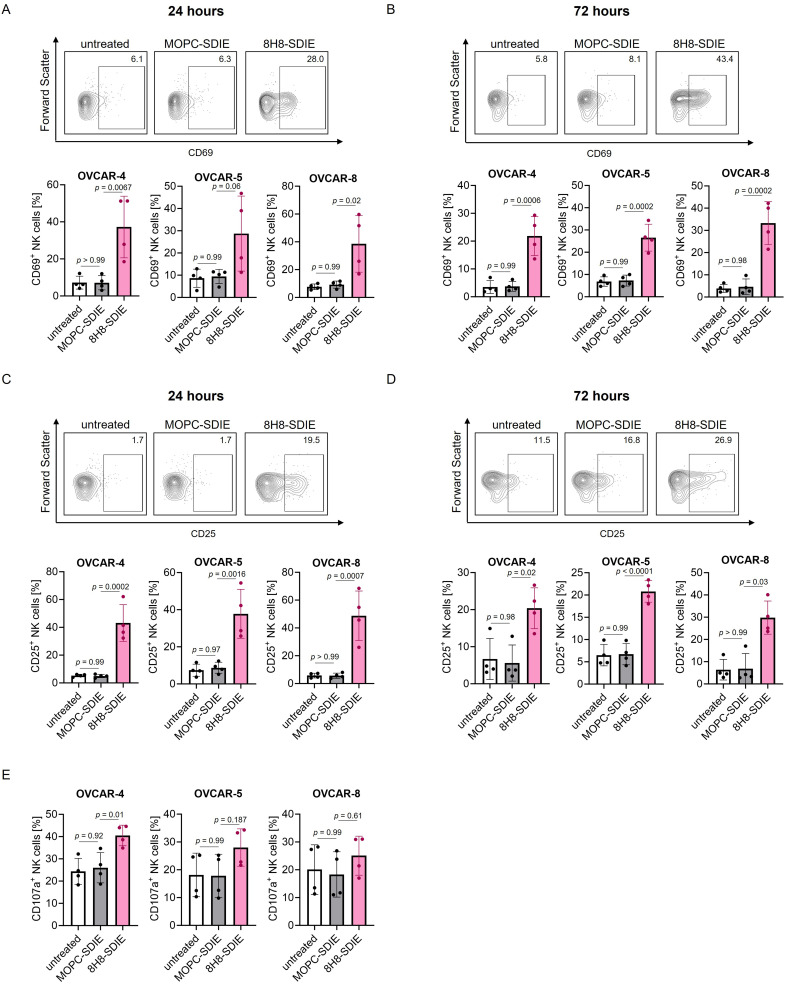
Induction of NK cell activation and immune synapse formation by 8H8-SDIE in the presence of ovarian cancer cells. PBMC of healthy donors were cultured with the indicated ovarian cancer cells at an E:T ratio of 2.5:1 in the presence or absence of 8H8-SDIE or MOPC-SDIE (1 µg/mL). The activation of NK cells, identified as CD56^+^CD3^−^ cells within PBMC, was determined after (**A**) 24 hours and (**B**) 72 hours for CD69, as well as the analysis of CD25 expression after (**C**) 24 hours and (**D**) 72 hours by flow cytometry. The top panels illustrate exemplary results obtained in a single experiment with PBMC of a single donor, the bottom panels depict combined analyses of data obtained with PBMC from four independent donors. Statistical significance was calculated by one-way ANOVA and Tukey’s multiple comparisons test (**A**–**D**) or Kruskal–Wallis test and Dunn’s multiple comparisons test (**D**). (**E**) Degranulation of CD56^+^CD3^−^ NK cells was assessed by flow cytometry analysis of CD107a expression after 4 hours. Shown are combined results from four independent PBMC donors with indicated ovarian cancer cell lines. Statistical significance was calculated by one-way ANOVA and Tukey’s multiple comparisons test. Bars with mean ± SD.

To ascertain the capacity of 8H8-SDIE to elicit NK cell reactivity against ovarian cancer cell lines, PBMC were cultured with the indicated cell lines in the presence or absence of 8H8-SDIE or the respective isotype control, MOPC-SDIE. Following a 24 hour and 72 hour incubation period, the expression of activation markers CD69 and CD25 on NK cells within PBMC was analyzed by flow cytometry. After treatment with 8H8-SDIE, expression of both activation markers was significantly increased at both analyzed time points (Figure 4A–D). The analysis of NK cell degranulation within PBMC, as determined by the examination of CD107a expression, confirmed that 8H8-SDIE activated NK cells (Figure 4E). No effects were observed when treating with MOPC-SDIE (Figure 4A–E).

**Figure 5:**
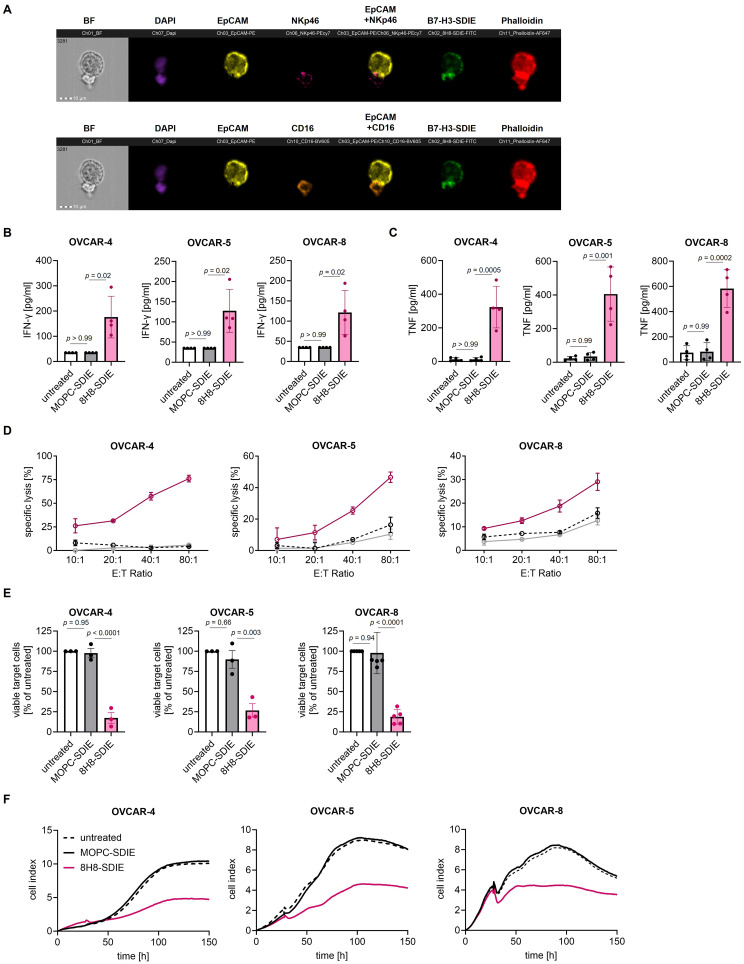
NK cell reactivity and cytotoxicity against ovarian cancer upon treatment with 8H8-SDIE. (**A**) NK cells from healthy donors were incubated with OVCAR-4 tumor cells at an E:T ratio of 1:1 in the presence of 8H8-SDIE (1 µg/mL). The colocalization and formation of immune synapses was analyzed after two hours of incubation using amnis ImageStream mk II. Cells were stained with fluorescent-labeled antibodies. Fluorescent-labeled Phalloidin was added after fixation, and DAPI was used for counterstaining. The colocalization of ovarian cancer cells and NK cells was analyzed by gating on doublets. Immune synapse formation between NK and target cell doublets was analyzed by measuring colocalization in the overlapping region of polarized Phalloidin. The scale bar is 10 µm. BF = bright field. (**B**–**F**) PBMC of healthy donors were cultured with the indicated ovarian cancer cells in the presence or absence of 8H8-SDIE or MOPC-SDIE (1 µg/mL). (**B** and **C**) Cells were cultured for 4 hours with an E:T ratio of 2.5:1, then supernatants were analyzed for (**B**) IFN-γ and (**C**) TNF by Legendplex assays. Statistical significance was calculated by Kruskal–Wallis test and Dunn’s multiple comparisons test (**B**) or one-way ANOVA and Tukey’s multiple comparisons test (**C** and **D**) Tumor cell lysis was measured after 2 hours using Europium-based cytotoxicity assays. Shown are exemplary results for ovarian cancer cell lines OVCAR-4, OVCAR-5 and OVCAR-8 with PBMC from one donor out of data obtained with different independent PBMC donors (n = 3–5). (**E**) Cell lysis of depicted ovarian cancer cells was determined at 72 hours using a FACS-based assay. Shown are combined data obtained with PBMC from three independent donors, E:T Ratio 10:1, untreated condition was defined as 100% viable target cells. Statistical significance was calculated by one-way ANOVA and Tukey’s multiple comparisons test. (**F**) The long-term cytotoxic effects of PBMC from four independent donors against ovarian cancer cells were determined using the xCELLigence system, E:T ratio 10:1. Data are presented as mean ± SD.

Subsequently, we examined whether 8H8-SDIE facilitates the colocalization of NK cells and tumor cells, as well as the formation of an immune synapse. Isolated NK cells were incubated with OVCAR-4 cells and 8H8-SDIE. The colocalization of tumor and isolated NK cells was analyzed by gating on doublets, and the binding of 8H8-SDIE was visualized by staining with a fluorescence-conjugated anti-human mAb. Immune synapse formation was determined by Phalloidin staining. The results demonstrated that 8H8-SDIE bound tumor cells in all colocalized cases, leading to the formation of an immune synapse (Figure 5A, Supplemental Figure S2).

### 8H8-SDIE Induces NK Cell Reactivity Against Ovarian Cancer Cells

We next analyzed whether NK cell activation by 8H8-SDIE was accompanied by a corresponding effect on cytokine release. To this end, PBMC were co-cultured with the indicated target cells and treated with 8H8-SDIE or MOPC-SDIE. A Legendplex assay of culture supernatants after 4 hours revealed a significant increase in the immune effector cytokines IFN-γ (Figure 5B) and TNF (Figure 5C), while the isotype control exhibited no effect. Finally, we evaluated the capacity of 8H8-SDIE to induce ADCC which results in lysis of ovarian cancer cells. The results demonstrated that 8H8-SDIE markedly induced target cell lysis in short-term ADCC assays across all cell lines examined, irrespective of the levels of B7-H3 expression in these cell lines. In contrast, the presence of control mAb had no discernible impact on target cell lysis (Figure 5C). Furthermore, analyses using flow cytometry-based lysis assays over 72 hours demonstrated the marked efficacy of 8H8-SDIE against ovarian cancer cells (Figure 5D). Finally, to ascertain whether 8H8-SDIE exerts cytotoxic effects over extended periods of time, which is necessary for sustained efficacy, long-term killing assays using PBMC were conducted using the xCELLigence system for 120 hours (Figure 5E). Despite the differences in B7-H3 surface expression, 8H8-SDIE induced tumor cell killing in all three cell lines utilized.

## Discussion

Ovarian cancer remains one of the most lethal gynecologic cancers. Although notable progress has been achieved in recent years - particularly with the introduction of PARP inhibitors, as demonstrated by the SOLO1 trial,^10^ and ADCs such as mirvetuximab soravtansine-gynx in the MIRASOL trial^17^ - these therapies are currently applicable only to selected patient subgroups. Moreover, a considerable number of patients are diagnosed at an advanced stage, often exhibiting evidence of metastatic disease.^53^ Additionally, a considerable obstacle is the high rate of relapse following the completion of primary tumor therapy. While current advancements have significantly improved outcomes for some patients, a substantial proportion continues to experience limited long-term benefit. Therefore, there remains a significant medical need for the development of more efficacious, low-toxicity maintenance therapies and for the identification of novel approaches that can be offered to a broader patient population in relapse, particularly for patients who do not qualify for existing targeted options.^54^ Consequently, there is a compelling need for the development of novel treatment modalities.^55^ Antibodies have emerged as a proven and rapidly expanding modality for treating human diseases, including cancer, due to their high specificity for their targets and favorable safety profiles. Notably, this field reached a significant milestone recently, with the 100th therapeutic antibody being approved by the FDA in 2021.^56^

B7-H3 (CD276), a member of the B7 family, is regarded as a promising candidate for immunotherapy in cancer treatment. There is mounting evidence that B7-H3 is highly expressed in a number of tumor types and plays a role in immune suppression and tumor progression.^57^ In ovarian cancer, B7-H3 has been demonstrated to be expressed independently of BRCA deficiency, suggesting that targeting this molecule could be an efficacious strategy for the majority of patients.^41^ Moreover, B7-H3 has been shown to be elevated in ovarian tumor tissues from patients with aggressive tumors and was linked to stem-like phenotypes and drug resistance.^58^ Of particular note is the observation that in the ovarian tumor microenvironment (TME), stromal cells express B7-H3 at high levels, which allows for targeting of fibroblasts, which have been demonstrated to possess immunosuppressive activities.^36^ Given that B7-H3 is not only expressed on tumor cells and the TME but also on the tumor vasculature in various malignancies, targeting B7-H3 has the potential to facilitate enhanced infiltration of immune effector cells into solid tumors.^59^

Following the analysis of tissue sections from 43 ovarian cancer patients, including two different intraabdominal tumor localizations for some patient samples, it was observed that membranous staining for B7-H3 was present in all specimen. Moreover, the expression was observed to extend to the tumor-surrounding structures including stromal tissue, blood vessels, and capillaries adjacent to the tumor cells in a considerable number of cases. The consistent detection of B7-H3 across all HGSC samples likely reflects its broad distribution not only on tumor cells but also within the tumor microenvironment.^41^ While this is in line with the previous reports,^60^ we acknowledge that our sample size may not fully capture the range of expression heterogeneity in HGSC. In light of these findings and the previously discussed role of B7-H3, we conclude that a B7-H3 mAb would be a promising immunotherapy for the treatment of ovarian cancer. Our findings revealed that treatment with 8H8-SDIE resulted in the formation of tumor-NK cell synapses, notable NK cell activation, and cytokine release, ultimately leading to significant tumor cell lysis in both short- and long-term in vitro settings. From a clinical perspective, B7-H3 targeting may represent a promising therapeutic option for HRD-negative ovarian cancer patients, who currently have limited treatment alternatives, and may further enhance efficacy by addressing B7-H3-expressing components of the tumor microenvironment, such as tumor vasculature and cancer-associated fibroblasts.

NK cells have a unique advantage in cancer immunotherapy: their ability to target and eliminate tumor cells without prior sensitization and their ability to induce ADCC.^61^ Accordingly, NK cells have received considerable attention for immunotherapies, including both cellular and pharmaceutical approaches. Application of antitumor antibodies which induce ADCC represents a promising therapeutic approach for many cancers, as demonstrated for example by the clinical success of rituximab.^62^ Notably, to date, no NK cell-based immunotherapeutic compounds have been approved for the treatment of ovarian cancer patients, despite the fact that a number of preclinical studies have demonstrated that ovarian cancer cells are susceptible to attack by NK cells.^63–65^ Given that the induction of ADCC represents a significant effector mechanism mediated by antitumor mAbs, considerable attention is currently being devoted to enhancing the efficacy of antitumor mAbs by increasing the affinity of the Fc region to CD16, which is expressed on the surface of NK cells and the majority of gamma delta (γδ) T cells.^66^ In addition to the modification of glycosylation motifs,^67^ increased affinity to CD16 can be achieved by altering the amino acid sequence in the CH2 domain of the Fc region. An example of this is the S239D/I332E substitution (SDIE modification), which is also present in our B7-H3 targeting construct 8H8-SDIE.^30^ Notably, the SDIE modification is also carried by clinically approved mAbs, including the HER2 mAb margetuximab^68^ and the CD19 mAb tafasitamab.^69^ These antibodies show enhanced binding to activating Fcγ receptors, including FcγRI (CD64) and FcγRIIIa (CD16), leading to improved effector functions such as ADCC but also ADCP.^70^ Importantly, clinical data from tafasitamab indicate that the SDIE modification is well tolerated and does not result in significant off-target effects, supporting its use in therapeutic antibodies like 8H8-SDIE. Targeting of both NK and γδ T cells may be a relevant consideration for immunotherapy with Fc-enhanced mAbs. It has been demonstrated that treatment with tafasitamab not only markedly improves NK cell ADCC, but also induces target cell lysis by γδ T cells.^71^ Verification of this benefit aspect may be conducted in further studies with the 8H8-SDIE mAb.

When using therapeutic antibodies, the TME is a critical factor influencing therapeutic efficacy. The ovarian TME is particularly complex and presents multiple therapeutic challenges. Features such as dysfunctional vasculature, elevated interstitial fluid pressure, and widespread immunosuppression can significantly limit treatment success.^72^,^73^ Additionally, immunosuppressive components of the TME, including regulatory T cells (Tregs), myeloid-derived suppressor cells (MDSCs), and tumor-associated macrophages,^74^ were not addressed in our current experimental systems. Therefore, future preclinical studies incorporating in vivo models that more closely mimic the immunosuppressive conditions of the patient tumor microenvironment are warranted to better evaluate therapeutic efficacy.

A significant challenge to antibody-based immunotherapy is the expression of target antigens on healthy tissues and cells, which may lead to adverse effects. It is important to note that B7-H3 is expressed on a number of different cell types, including antigen-presenting cells, endothelial cells, resting fibroblasts, amniotic fluid stem cells, and osteoblasts. Additionally, low B7-H3 expression was observed in healthy liver tissue.^59^ In the TAMARACK clinical trial (NCT 05551117) utilizing B7-H3 as a target antigen for the ADC vobramitamab duocarmazine, efficacy was demonstrated and the preliminary results from the ongoing Phase 1/2 clinical trial in patients with metastatic castration-resistant prostate cancer suggest encouraging antitumor activity of a B7-H3-targeting ADC, supporting continued investigation.^75^,^76^ However adverse events were also documented in a subset of patients.^76^ It is plausible to hypothesize that these events may have occurred due to the elevated toxicity of an ADC in comparison to mAbs. Nevertheless, recent clinical advances highlight the growing therapeutic relevance of B7-H3 as a target in solid tumors. Promising results have been reported for B7-H3-directed antibody-drug conjugates, such as YL201 and HS-20093, with notable objective response rates across multiple tumor types.^77^,^78^ Early clinical signals have also led to FDA Breakthrough Therapy Designation for agents such as GSK5764227. In addition, preclinical investigations employing therapeutic strategies targeting B7-H3, including B7-H3-targeting CAR T cells, and novel combination approaches such as B7-H3-targeting bsAbs and immune checkpoint blockade have demonstrated a notable anti-tumor effect in experimental models,^79^ despite the weak expression of B7-H3 on certain non-tumor cells.^33^,^80^,^81^ Furthermore, the safety profile of an mAb is anticipated to be superior to that of T cell-based approaches, as NK cells release less pro-inflammatory cytokines, which can potentially lead to cytokine release syndrome, compared to T cells.^82–84^ Although B7-H3 is expressed at low levels in certain healthy tissues,^32^ its widespread overexpression in tumors, together with the promising preclinical and clinical results of immunotherapeutic strategies targeting B7-H3 and the favorable safety profile of NK cell-engaging mAbs, highlight the potential of our B7-H3 SDIE as a therapeutic target in solid tumors, but still require careful evaluation, particularly in in vivo models.

## Conclusion

In conclusion, the preclinical data reported in this study, which document the efficacy of 8H8-SDIE in ovarian cancer, in our view clearly indicates that 8H8-SDIE constitutes a promising novel option for ovarian cancer treatment.

## Data Availability

The datasets supporting the conclusions of this article are included within the article. Part of the data was presented at the SITC Meeting 2024 and a congress abstract without data was published.^85^.

## Abbreviations

7-AAD, 7-aminoactinomycin D; ADC, antibody-drug-conjugate; ADCC, antibody dependent cellular cytotoxicity; bsAb, bispecific antibody; CD, cluster of differentiation; Dmmr, (deficient mismatch repair; ELISA, enzyme-linked immunosorbent assay; E:T, effector-to-target; EU, endotoxin; FACS, fluorescent activated cell sorting; FBS, fetal bovine serum; FcγR, Fc gamma receptor; FDA, Food and Drug Administration; FFPE, formalin-fixed and paraffin-embedded; γδ, gamma delta; H&E, hematoxylin and eosin; HER2, human epidermal growth factor receptor 2; HGSC, high-grade serous carcinoma; IFN-γ, Interferon γ; IgG1, Immunoglobulin G1; mAb, monoclonal antibody; MFI, median fluorescence intensity; MSI-H, microsatellite instability-high; NK, natural killer; ns, not significant; PARP, Poly-ADP-ribose polymerase; PBMC, peripheral blood mononuclear cells; PCR, polymerase chain reaction; PE, phycoerythrin; RT, room temperature; SD, standard deviation; TMA, tissue microarray; TME, tumor microenvironment; TMB-H, high tumor mutational burden; TNF, tumor necrosis factor; WT, wildtype.
